# Minimally invasive surgery versus open gastrectomy for older patients with gastric cancer: A propensity score‐matching analysis

**DOI:** 10.1002/ags3.12842

**Published:** 2024-07-15

**Authors:** Masaaki Yamamoto, Takeshi Omori, Yasunori Masuike, Naoki Shinno, Hisashi Hara, Takahito Sugase, Takashi Kanemura, Atsushi Takeno, Motohiro Hirao, Hiroshi Miyata

**Affiliations:** ^1^ Department of Gastroenterological Surgery Osaka International Cancer Institute 3‐1‐69 Otemae, Chuo‐ku Osaka 5418567 Osaka Japan; ^2^ Department of Surgery NHO Osaka National Hospital 2‐1‐14 Hoenzaka, Chuo‐ku Osaka 5400006 Osaka Japan

**Keywords:** elderly, gastrectomy, gastric cancer, general surgery, minimally invasive surgery

## Abstract

**Aim:**

To compare minimally invasive and open surgery for older patients with gastric cancer.

**Methods:**

This study included 464 consecutive patients with gastric cancer aged ≥75 years who underwent open or laparoscopic gastrectomy at our institution from January 2004 to December 2018. We performed propensity score‐matching and compared short‐ and long‐term outcomes between the two groups.

**Results:**

After matching, 332 patients were included in the study (166 in each group). The laparoscopy group had a longer operative time, lesser blood loss, and shorter hospital stays than the open surgery group (all *p* < 0.020). The laparoscopy group had a lower complication rate than the open surgery group (*p* = 0.002). No significant differences were noted in the 3‐y overall, recurrence‐free, and disease‐free survival between the groups (all *p* > 0.200).

**Conclusion:**

Minimally invasive surgery for older patients with gastric cancer may be more beneficial than open gastrectomy in terms of blood loss and hospital stay.

## Graphical abstract

This study aimed to compare minimally invasive and open surgery for older patients with gastric cancer. Minimally invasive surgery for older patients with gastric cancer may be more beneficial than open gastrectomy in terms of blood loss and hospital stay.

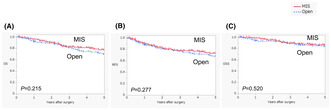

## 1 INTRODUCTION

Worldwide, the population is aging, resulting in an increase in the number of older patients with gastric cancer. As the standard treatment for gastric cancer is surgery, the number of surgeries performed on older patients with gastric cancer is increasing.

As individuals age, their physiological functions decline and their immune system tends to weaken. Older people commonly have a variety of chronic health issues, including those of the heart, lungs, and brain.^1^, ^2^ Therefore, the postoperative complication rate among older patients is higher than that among younger patients.^3^, ^4^, ^5^, ^6^, ^7^

Laparoscopic distal gastrectomy (LDG) with D2 lymphadenectomy for locally advanced gastric cancer has proved noninferior to open distal gastrectomy (ODG) in terms of short‐ and long‐term outcomes.^8^, ^9^, ^10^ As minimally invasive surgery (MIS) is less invasive than open surgery, it may be beneficial for older patients with various comorbidities. However, few reports on comparisons of short‐ and long‐term outcomes between MIS and open surgery for older patients with gastric cancer have been published.^11^ That was the aim of the present study.

## 2 METHODS

### 2.1 Patients

We retrospectively collected data of consecutive patients who underwent radical gastrectomy for gastric cancer at the Osaka International Cancer Institute from January 2004 to December 2018. The following inclusion criteria were used: age ≥75 years, histologically diagnosed adenocarcinoma of the stomach, having sufficient data for analysis, and not having a clinical M stage. Tumor status (TNM staging) was evaluated based on the 15th edition of the Japanese Classification of Gastric Carcinoma,^12^ which is equivalent to the 8th edition of the Union for International Cancer Control/TNM Classification.^13^ We evaluated complications by using the Clavien–Dindo classification.^14^ We evaluated the comorbidities by using the Charlson Comorbidity Index.^15^

The protocol for this research project was approved by the Ethics Review Committee of the Osaka International Cancer Institute (approval number #23151) and conforms to the provisions of the Declaration of Helsinki. All informed consent was obtained from the participants.

### 2.2 Surgery

All patients underwent open gastrectomy (open surgery group) or laparoscopic gastrectomy (MIS group) with regional lymphadenectomy based on the Japanese Gastric Cancer Treatment Guidelines, 5th edition.^16^ The procedure followed for laparoscopic gastrectomy was previously described.^17^ To ensure the quality of surgery in Japan, the Endoscopic Surgical Skill Qualification System (ESSQS) was established by the Japan Society for Endoscopic Surgery.^18^ All surgeries were performed by or under the direction of board‐certified surgeons with ESSQS certification.

### 2.3 Postoperative management

Postoperatively, patients were routinely followed up (3–6 mo) based on the Japanese Gastric Cancer Treatment Guidelines, 5th edition.^16^

### 2.4 Propensity score matching

We conducted a propensity score‐matching (PSM) analysis to balance the groups based on the following covariates: age, sex, body mass index, American Society of Anesthesiologists physical status, Charlson Comorbidity Index, clinical T stage, clinical N stage, and type of resection. The propensity score was estimated using a logistic regression model with a greedy‐matching algorithm without replacement and with a caliper of 0.2 standard deviations.

### 2.5 Statistical analysis

Associations between clinicopathological factors and the open or MIS group were analyzed using the chi‐square test for categorical variables and the Mann–Whitney *U* test for continuous variables. Disease‐specific survival (DSS) was defined as the period from the date of surgery to the date of death from gastric cancer, including operative mortality. Recurrence‐free survival (RFS) was defined as the period from the date of surgery to the date of detection of the first recurrence or death from any cause. Overall survival (OS) was defined as the period from the date of surgery to the date of death from any cause. The Kaplan–Meier method was used for survival analysis, and differences in survival were tested using the log‐rank test. All statistical comparisons were two‐sided, and *p* < 0.05 was considered statistically significant. All statistical analyses were performed using JMP Pro 17.0.0 software (SAS Institute, Cary, NC, USA).

## 3 RESULTS

### 3.1 Patient characteristics

**FIGURE 1:**
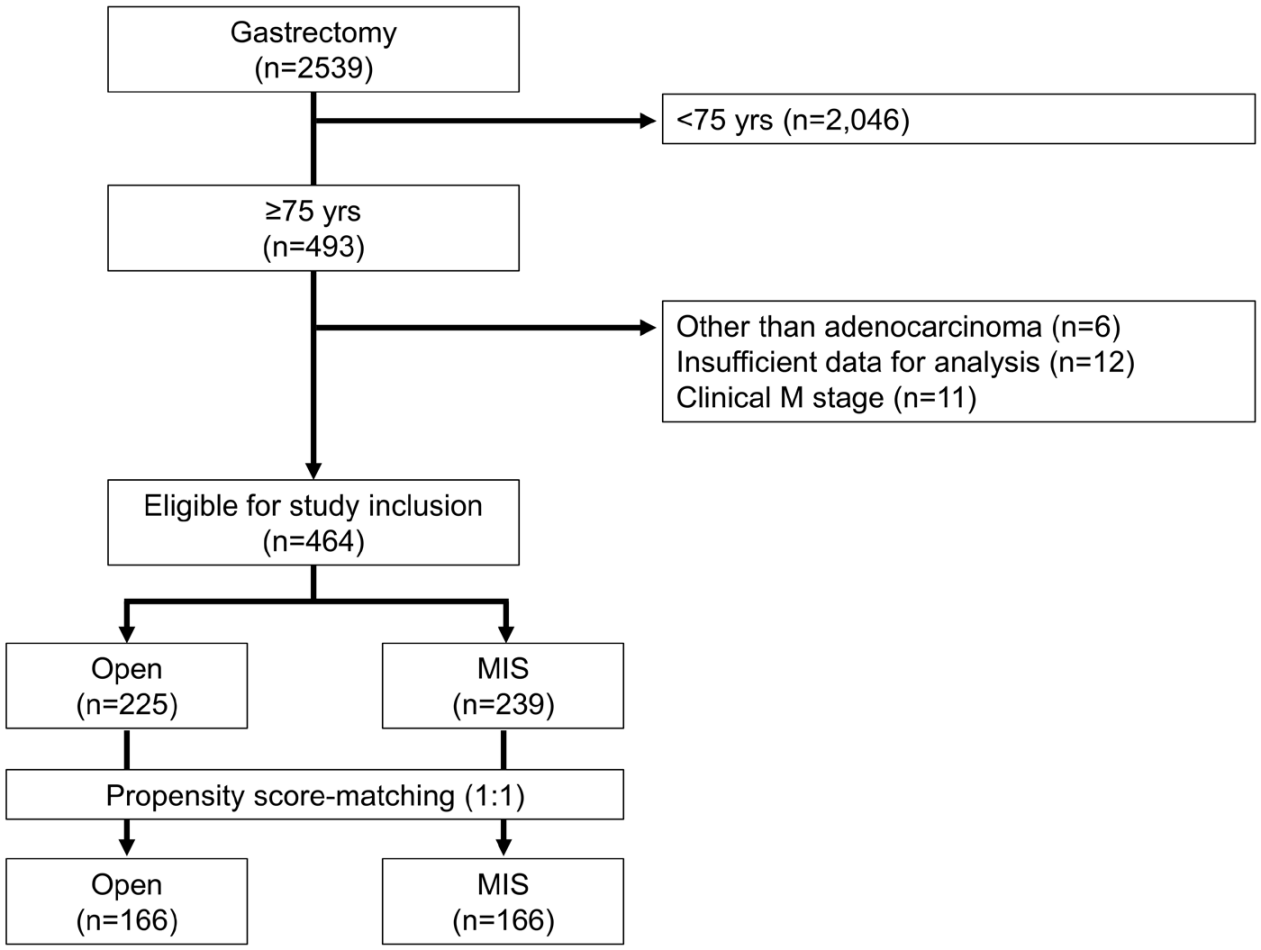
Flowchart of patient selection for the study. MIS, minimally invasive surgery; Open, open surgery.

We initially screened 2539 patients from our database who had undergone gastrectomy. After applying the inclusion criteria, 464 patients (225 in the open surgery group and 239 in the MIS group) were included for analysis. After PSM, 332 patients were included in the study (166 in each group). A flowchart of this process is presented in Figure 1. The patients' clinical characteristics before and after PSM are summarized in Table 1.

**TABLE 1:** Patients' characteristics in open surgery and MIS groups before and after matching.

	Before matching			After matching		
	Open (*n* = 225)	MIS (*n* = 239)	*p*	Open (*n* = 166)	MIS (*n* = 166)	*p*
Age (years, mean ± SD)	79.1 ± 3.6	79.2 ± 3.8	0.961	79.0 ± 3.8	78.8 ± 3.5	0.640
Age (years)						
75–79	145 (64.4%)	151 (63.2%)	0.550	109 (65.7%)	112 (67.5%)	0.894
80–84	63 (28.0%)	63 (26.4%)		43 (25.9%)	42 (25.3%)	
85–89	14 (6.2%)	20 (8.4%)		11 (6.6%)	10 (6.0%)	
90–94	2 (0.9%)	5 (2.1%)		2 (1.2%)	2 (1.2%)	
95–99	1 (0.4%)	0 (0%)		1 (0.6%)	0 (0%)	
Sex						
Male	167 (74.2%)	159 (66.5%)	0.070	118 (71.1%)	116 (69.9%)	0.810
Female	58 (25.8%)	80 (33.5%)		48 (28.9%)	50 (30.1%)	
BMI (kg/m^2^, mean ± SD)	22.5 ± 2.9	22.2 ± 3.1	0.190	22.4 ± 3.0	22.5 ± 3.0	0.897
ASA‐PS score						
1, 2	171 (76.0%)	176 (73.6%)	0.560	124 (74.7%)	125 (75.3%)	0.899
3	54 (24.0%)	63 (26.4%)		42 (25.3%)	41 (24.7%)	
Charlson comorbidity index						
<6	189 (84.0%)	194 (81.2%)	0.423	136 (81.9%)	136 (81.9%)	1.000
≥6	36 (16.0%)	45 (18.8%)		30 (18.1%)	30 (18.1%)	
Charlson comorbidity						
Myocardial infarction	25 (11.1%)	23 (9.6%)	0.599	18 (10.8%)	17 (10.2%)	0.858
Chronic heart failure	8 (3.6%)	6 (2.5%)	0.511	5 (3.0%)	3 (1.8%)	0.474
Peripheral vascular disease	6 (2.7%)	6 (2.5%)	0.916	5 (3.0%)	4 (2.4%)	0.735
Cerebrovascular disease	23 (10.2%)	21 (8.8%)	0.598	20 (12.0%)	16 (9.6%)	0.480
Dementia	1 (0.4%)	2 (0.8%)	0.598	0 (0%)	2 (1.2%)	0.156
Chronic pulmonary disease	18 (8.0%)	16 (6.7%)	0.590	13 (7.8%)	11 (6.6%)	0.672
Connective tissue disorder	4 (1.8%)	5 (2.1%)	0.806	4 (2.4%)	4 (2.4%)	1.000
Liver disease	2 (0.9%)	2 (0.8%)	0.952	2 (1.2%)	1 (0.6%)	0.562
Diabetes mellitus	41 (18.2%)	54 (22.6%)	0.244	34 (20.5%)	37 (22.3%)	0.688
Chronic kidney failure	1 (0.4%)	4 (1.7%)	0.200	1 (0.6%)	3 (1.8%)	0.314
Other tumor/lymphoma	9 (4.0%)	14 (5.9%)	0.357	8 (4.8%)	10 (6.0%)	0.628
Histological type (dominant)						
Differentiated type	149 (66.2%)	158 (66.1%)	0.979	116 (69.9%)	113 (68.1%)	0.722
Undifferentiated type	76 (33.8%)	81 (33.9%)		50 (30.1%)	53 (31.9%)	
cT						
cT1	87 (38.7%)	151 (63.2%)	<0.001	87 (52.4%)	88 (53.0%)	0.999
cT2	56 (24.9%)	32 (13.4%)		28 (16.9%)	28 (16.9%)	
cT3	43 (19.1%)	35 (14.6%)		30 (18.1%)	30 (18.1%)	
cT4	39 (17.3%)	21 (8.8%)		21 (12.7%)	20 (12.0%)	
cN						
cN0	140 (62.2%)	193 (80.8%)	<0.001	123 (74.1%)	121 (72.9%)	0.804
cN+	85 (37.8%)	46 (19.2%)		43 (25.9%)	45 (27.1%)	
cM						
cM0	225 (100%)	239 (100%)	–	166 (100%)	166 (100%)	–
cM1	0 (0%)	0 (0%)		0 (0%)	0 (0%)	
cStage						
cStage I	116 (51.6%)	171 (71.5%)	<0.001	105 (63.3%)	105 (63.3%)	0.873
cStage II	49 (21.8%)	34 (14.2%)		26 (15.7%)	27 (16.3%)	
cStage III	55 (24.4%)	32 (13.4%)		31 (18.7%)	32 (19.3%)	
cStage IV	5 (2.2%)	2 (0.8%)		4 (2.4%)	2 (1.2%)	
Procedure						
Total gastrectomy	86 (38.2%)	46 (19.2%)	<0.001	49 (29.5%)	46 (27.7%)	0.762
Distal gastrectomy	122 (54.2%)	161 (67.4%)		101 (60.8%)	100 (60.2%)	
Proximal gastrectomy	17 (7.6%)	32 (13.4%)		16 (9.6%)	20 (12.0%)	
pT						
pT1	97 (43.1%)	142 (59.4%)	0.002	87 (52.4%)	84 (50.6%)	0.417
pT2	26 (11.6%)	29 (12.1%)		15 (9.0%)	23 (13.9%)	
pT3	53 (23.6%)	37 (15.5%)		38 (22.9%)	30 (18.1%)	
pT4	49 (21.8%)	31 (13.0%)		26 (15.7%)	29 (17.5%)	
pN						
pN0	126 (56.0%)	151 (63.2%)	0.134	104 (62.7%)	97 (58.4%)	0.862
pN1	34 (15.1%)	40 (16.7%)		25 (15.1%)	26 (15.7%)	
pN2	30 (13.3%)	26 (10.9%)		19 (11.4%)	23 (13.9%)	
pN3	35 (15.6%)	22 (9.2%)		18 (10.8%)	20 (12.0%)	
pStage						
pStage I	108 (48.0%)	152 (63.6%)	<0.001	94 (56.6%)	92 (55.4%)	0.399
pStage II	50 (22.2%)	46 (19.2%)		33 (19.9%)	37 (22.3%)	
pStage III	62 (27.6%)	32 (13.4%)		35 (21.1%)	28 (16.9%)	
pStage IV	5 (2.2%)	9 (3.8%)		4 (2.4%)	9 (5.4%)	

Abbreviations: ASA‐PS, American Society of Anesthesiologists physical status; BMI, body mass index; cStage, clinical stage; MIS, minimally invasive surgery; Open, open surgery; pStage, pathological stage; SD, standard deviation.

Before matching, the open surgery group statistically included a larger proportion of patients with advanced cT stage, cN stage, clinical stage, pT stage, and pathological stage (*p* < 0.001, *p* < 0.001, *p* < 0.001, *p* = 0.002, and *p* < 0.001, respectively). The rates of different types of gastrectomy also significantly differed between the open surgery and MIS groups (*p* < 0.001). After matching, no significant differences were observed in the clinical characteristics between the groups.

We examined the association between the open and MIS groups and operative factors (Table 2). After matching, the operative time in the MIS group was longer than that in the open surgery group (*p* = 0.019). However, blood loss and the number of blood transfusions were significantly lower in the MIS group (both *p* < 0.001). The hospital stay after surgery and the total hospital stay in the MIS group were significantly shorter than those in the open surgery group (both *p* < 0.001).

**TABLE 2:** Intra‐ and postoperative factors in open surgery and MIS groups before and after matching.

	Before matching			After matching		
	Open (*n* = 225)	MIS (*n* = 239)	*p*	Open (*n* = 166)	MIS (*n* = 166)	*p*
Operative time (min), median (range)	230 (125–515)	241 (98–531)	0.230	228.5 (125–515)	248.5 (98–531)	0.019
Blood loss (mL), median (range)	420 (0–4200)	10 (0–4755)	<0.001	380 (0–4200)	10 (0–745)	<0.001
Blood transfusion, *n* (%)	53 (23.6%)	14 (5.9%)	<0.001	34 (20.5%)	10 (6.0%)	<0.001
Hospital stay after surgery (d), median (range)	15 (7–160)	9 (4–61)	<0.001	15 (7–115)	9 (6–58)	<0.001
Total hospital stay (d), median (range)	22 (9–170)	12 (7–67)	<0.001	21 (10–123)	12 (8–62)	<0.001

Abbreviations: MIS, minimally invasive surgery; Open, open surgery.

### 3.2 Postoperative complications

Postoperative complications are presented in Table 3. After PSM, the total rate of postoperative complications in the open surgery group was higher than that in the MIS group (*p* = 0.002). Of note, surgical site infections were more prevalent in the open surgery group (*p* = 0.010).

**TABLE 3:** Complications in open surgery and MIS groups before and after matching.

	Before matching			After matching		
	Open (*n* = 225)	MIS (*n* = 239)	*p*	Open (*n* = 166)	MIS (*n* = 166)	*p*
Complication (≥CD II)			<0.001			0.002
No	164 (72.9%)	215 (90.0%)		127 (76.5%)	148 (89.2%)	
Yes	61 (27.1%)	24 (10.0%)		39 (23.5%)	18 (10.8%)	
Pancreatic fistula	14 (6.2%)	3 (1.3%)	0.004	6 (3.6%)	3 (1.8%)	0.311
Surgical site infection	12 (5.3%)	1 (0.4%)	0.001	9 (5.4%)	1 (0.6%)	0.010
Abdominal abscess	10 (4.4%)	3 (1.3%)	0.038	6 (3.6%)	3 (1.8%)	0.311
Anastomotic leakage	7 (3.1%)	5 (2.1%)	0.490	4 (2.4%)	3 (1.8%)	0.703
Pneumonia	7 (3.1%)	3 (1.3%)	0.169	4 (2.4%)	3 (1.8%)	0.703
Delayed gastric emptying	5 (2.2%)	2 (0.8%)	0.221	4 (2.4%)	2 (1.2%)	0.410
Bowel obstruction	4 (1.8%)	3 (1.3%)	0.644	3 (1.8%)	2 (1.2%)	0.652
Bleeding	4 (1.8%)	3 (1.3%)	0.644	3 (1.8%)	2 (1.2%)	0.652
Delirium	3 (1.3%)	1 (0.4%)	0.287	2 (1.2%)	1 (0.6%)	0.562
Arrhythmia	2 (0.9%)	1 (0.4%)	0.527	2 (1.2%)	1 (0.6%)	0.562
Anastomotic stenosis	2 (0.9%)	1 (0.4%)	0.527	0 (0%)	0 (0%)	–
Others	13 (5.8%)	2 (0.8%)	0.003	7 (4.2%)	1 (0.6%)	0.032

Abbreviations: CD, Clavien–Dindo class; MIS, minimally invasive surgery; Open, open surgery.

### 3.3 Pre‐ and postoperative chemotherapy

None of the patients had received preoperative chemotherapy. Regarding postoperative chemotherapy, 20 of 166 patients (12.0%) in the open group received adjuvant chemotherapy, of whom 19 (95%) received S‐1 or capecitabine alone. In the MIS group, 38 of 166 patients (22.9%) received adjuvant chemotherapy, of whom 36 (95%) received S‐1 or capecitabine alone.

### 3.4 Survival

**FIGURE 2:**
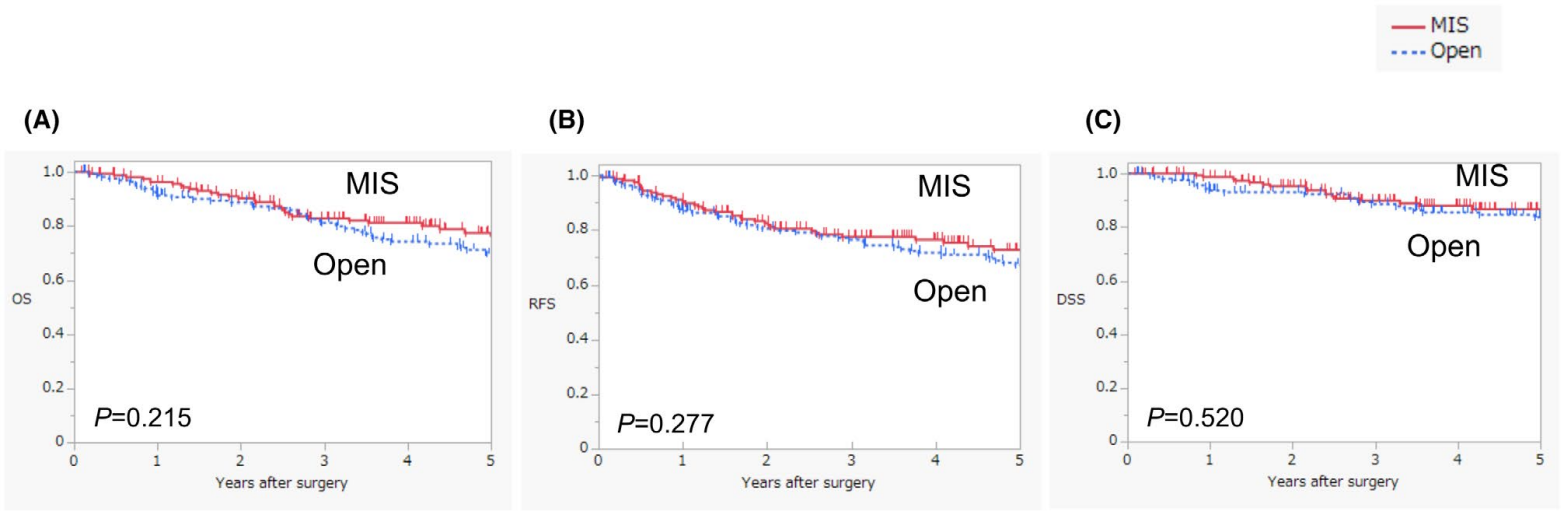
Comparison of survival curves between open surgery and MIS for overall survival(A), recurrence‐free survival(B), and disease‐free survival(C). DSS, disease‐specific survival; MIS, minimally invasive surgery; Open, open surgery; OS, overall survival; RFS, recurrence‐free survival.

The median follow‐up time for all patients was 52 mo. The 3‐y OS rates in the open surgery and MIS groups were 81.2% and 82.9%, respectively. No significant differences in OS were observed between the groups (log‐rank test, *p* = 0.215) (Figure 2A). The 3‐y RFS rates in the open surgery and MIS groups were 76.5% and 77.6%, respectively. No significant differences in RFS were observed between the groups (log‐rank test, *p* = 0.277) (Figure 2B). Regarding recurrence patterns, no significant differences were identified between the open surgery and MIS groups after PSM (Table 4). The 3‐y DSS rates in the open surgery and MIS groups were 88.6% and 89.8%, respectively. No significant differences in DSS were observed between the groups (log‐rank test, *p* = 0.520) (Figure 2C).

**TABLE 4:** Recurrence pattern in the open surgery and MIS groups before and after matching.

	Before matching			After matching		
	Open (*n* = 225)	MIS (*n* = 239)	*p*	Open (*n* = 166)	MIS (*n* = 166)	*p*
Recurrence	49 (21.8%)	30 (12.6%)	0.008	28 (16.9%)	26 (15.7%)	0.766
Peritoneal dissemination	16 (7.1%)	14 (5.9%)	0.583	10 (6.0%)	13 (7.8%)	0.517
Lymph nodes	8 (3.6%)	6 (2.5%)	0.511	5 (3.0%)	5 (3.0%)	1.000
Liver	13 (5.8%)	8 (3.3%)	0.208	8 (4.8%)	5 (3.0%)	0.396
Lungs	3 (1.3%)	4 (1.7%)	0.764	2 (1.2%)	4 (2.4%)	0.410
Local	6 (2.7%)	3 (1.3%)	0.271	5 (3.0%)	3 (1.8%)	0.474
Bone	3 (1.3%)	1 (0.4%)	0.287	1 (0.6%)	1 (0.6%)	1.000
Others	4 (1.8%)	2 (0.8%)	0.370	1 (0.6%)	2 (1.2%)	0.562

Abbreviations: MIS, minimally invasive surgery; Open, open surgery.

**FIGURE 3:**
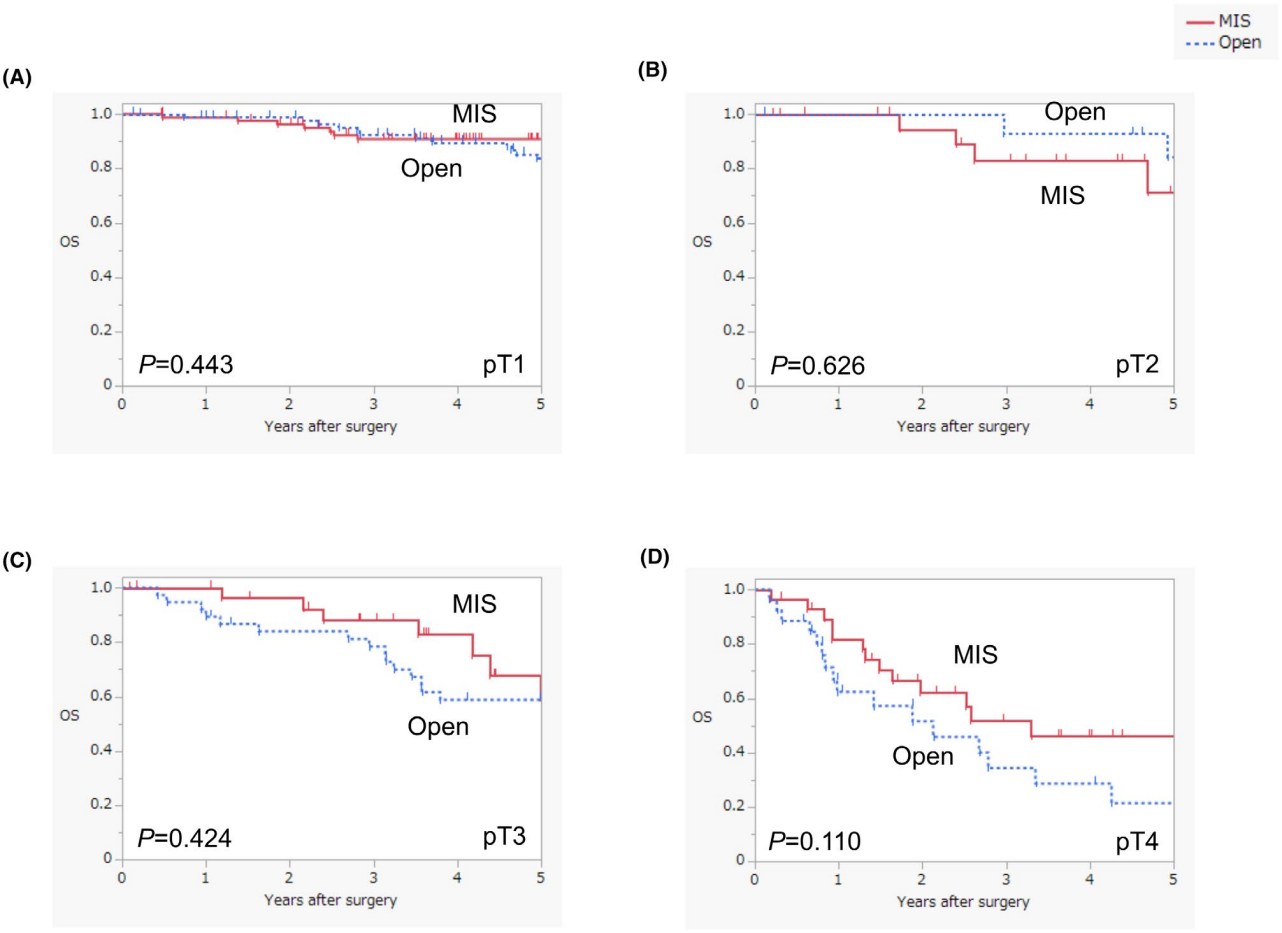
Comparison of survival curves between open surgery and MIS for overall survival according to pT stage(A; pT1, B; pT2, C; pT3, D; pT4). MIS, minimally invasive surgery; Open, open surgery; OS, overall survival.

**FIGURE 4:**
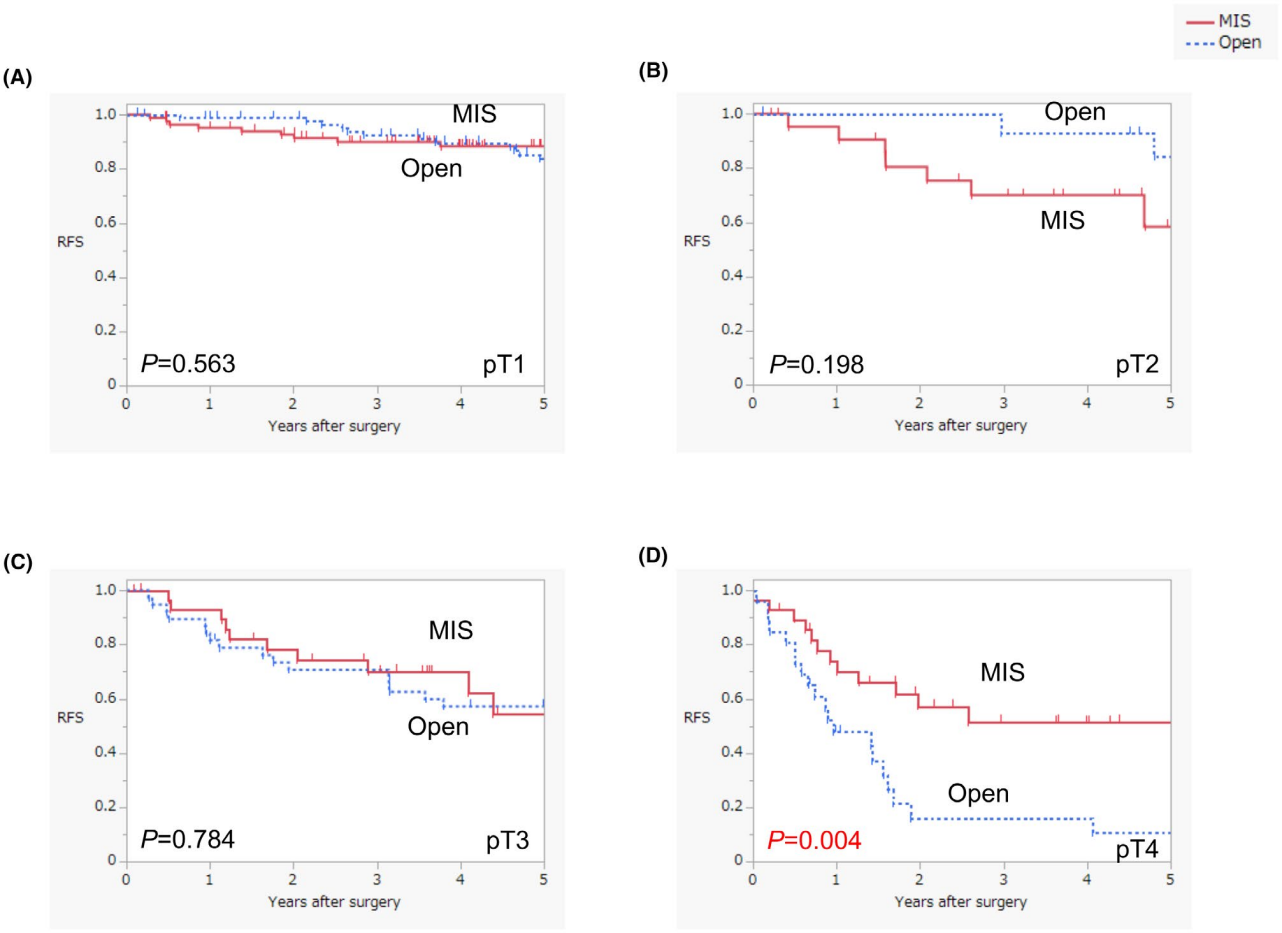
Comparison of survival curves between open surgery and MIS for recurrence‐free survival according to pT stage (A; pT1, B; pT2, C; pT3, D; pT4). MIS, minimally invasive surgery; Open, open surgery; RFS, recurrence‐free survival.

Subgroup analysis according to pT stage revealed no significant differences in OS between the open surgery and MIS groups (Figure 3). No significant differences were detected in RFS for patients with pT1, pT2, or pT3 disease between the groups (Figure 4A–C). The open surgery group had a significantly worse RFS than the MIS group among patients with pT4 disease (log‐rank test, *p* = 0.004) (Figure 4D). No significant differences were discovered with regard to DSS in patients with pT1, pT2, or pT3 disease between the groups (Figure S1A–C). The open surgery group had a seemingly worse DSS than the MIS group among patients with pT4 disease; however, this difference was not significant (log‐rank test, *p* = 0.068) (Figure S1D). The recurrence rate in the open surgery group was significantly higher than that in the MIS group among patients with pT4 disease (61.5% vs. 27.6%, *p* = 0.010) (Table S1).

In the PSM analysis of 332 older patients, 95 (28.6%) died during the study period: 61 (36.7%) in the open surgery group and 34 (20.5%) in the MIS group. In the open surgery group, 24 (39.3%) patients died of gastric cancer and 37 (60.7%) of other causes. The main breakdown of deaths from other diseases was 16 deaths from respiratory diseases, five from heart diseases, three from cerebrovascular diseases, and six from other cancers. In the MIS group, 18 (52.9%) patients died of gastric cancer and 16 (47.1%) of other causes. The proportion of deaths of other causes was higher in the open surgery group than that in the MIS group. The main breakdown of deaths from other diseases was five deaths from respiratory diseases, two from heart diseases, one from cerebrovascular disease, and three from other cancers.

Subgroup analysis according to pN stage revealed no significant differences with regard to OS, RFS, or DSS between the groups (Figures S2, S3A–C, and S4), except that the open group had a significantly worse RFS than the MIS group among patients with pN2 disease (log‐rank test, *p* = 0.029) (Figure S3D). Subgroup analysis according to pathological stage revealed no significant differences with regard to OS, RFS, or DSS between groups (Figures S5, S6A–C, and S7), except that the open surgery group had a significantly worse RFS than the MIS group among patients with pathological stage III disease (log‐rank test, *p* = 0.046) (Figure S6D).

## 4 DISCUSSION

Before this study, the usefulness of MIS in comparison with open gastrectomy for older patients with gastric cancer was unclear. In this study we revealed that short‐term outcomes in the MIS group were better than those in the open surgery group among patients aged ≥75 years, whereas the long‐term outcomes were equivalent between the groups. To our knowledge, this was the first large‐scale study in which PSM analysis was used to compare MIS and open gastrectomy among patients with gastric cancer aged ≥75 years.

The World Health Organization defines a person “65 years and older” as elderly. In contrast, in Japan, under the Act on Assuring Medical Care for the Elderly and its related laws, people aged 65 to 74 are defined as “early elderly,” and those aged 75 and older are defined as “late elderly.” Moreover, many studies on gastric cancer have been conducted specifically on patients aged ≥75 years.^19^, ^20^, ^21^, ^22^, ^23^, ^24^, ^25^ Therefore, we selected patients aged ≥75 years for this study.

Regarding short‐term outcomes in this study, MIS was superior to open surgery in terms of the complication rate, blood loss, hospital stay after surgery, and total hospital stay; the only exception was a slightly longer operative time. Although randomized controlled trials (RCTs) on patients in general indicated that MIS yields fewer complications and shorter hospital stays than open surgery, RCTs on older patients with gastric cancer in which MIS and open gastrectomy was compared have not been reported. Other, nonrandomized studies have indicated that laparoscopic gastrectomy for older patients yields a lower complication rate, less blood loss, and a shorter postoperative hospital stay than open gastrectomy.^11^, ^19^, ^21^ Older patients may benefit more from less‐invasive procedures than younger patients. Large scars caused by open surgery have a substantial impact on postoperative activities of daily living and necessitate a longer period of rehabilitation for a patient to return to the preoperative status than the small scars caused by laparoscopy. This difference is more pronounced in older patients than that in younger patients.

Regarding the long‐term outcomes in this study, no significant differences were observed between the MIS and open surgery groups in terms of OS, RFS, or DSS. The MIS group had significantly lower rates of recurrence than the open surgery group, although no significant difference in recurrence pattern was observed between the groups. Some RCTs of younger patients with gastric cancer revealed that LDG with D2 lymphadenectomy for locally advanced gastric cancer is noninferior to ODG in terms of short‐ and long‐term outcomes.^8^, ^9^, ^10^ Regarding older patients with gastric cancer, some investigators have reported that the OS after laparoscopic gastrectomy is similar to that after open gastrectomy.^26^, ^27^ Others have reported that both OS and DSS rates are significantly better following laparoscopic gastrectomy than those following open surgery in patients with gastric cancer, and that this effect was more pronounced among the older population.^21^ Yamamoto et al and Tanaka et al used PSM to compare open and laparoscopic surgeries among older patients who underwent gastrectomy.^11^, ^21^ Regarding the prognosis, Yamamoto et al reported that the OS and cancer‐specific survival in the open group was worse than those in the laparoscopic group. In contrast, Tanaka et al reported no significant differences in OS or DSS between the open and laparoscopic groups. In a recent meta‐analysis, the OS of older patients with gastric cancer who underwent laparoscopic gastrectomy was similar to that of patients who underwent open gastrectomy.^27^ Therefore, it seems that the OS of older patients with gastric cancer who undergo laparoscopic gastrectomy is similar to or better than that of older patients who undergo open gastrectomy. These results suggest that older patients with gastric cancer may be more amenable to the benefits of MIS.

In general, MIS is less invasive than open gastrectomy because the incision in MIS is much shorter. Moreover, MIS generally enables more precise surgery than open gastrectomy because of the magnified view it provides. Owing to these benefits, MIS may reduce patients' pain and blood loss, induce fewer complications, and yield shorter postoperative stays than open gastrectomy. Furthermore, single‐incision laparoscopic gastrectomy, which is truly MIS, reportedly yields a better prognosis than conventional multiport laparoscopic gastrectomy.^28^, ^29^ Moreover, the rate of complications of robotic gastrectomy, which is a kind of MIS that can be performed more precisely, is reportedly lower than that of laparoscopic gastrectomy.^30^ Therefore, as older patients are easily impacted by surgical invasion and have a higher risk of deteriorating organ function than younger patients, MIS may be the best option for such patients with gastric cancer.

Interestingly, in this study the subgroup analysis revealed that the prognosis (OS, RFS, and DSS) in the MIS group tended to be better than that in the open surgery group as the pT stage increased. In this study the recurrence rate in the open surgery group was significantly higher than that in the MIS group at pT4 (*p* = 0.011). Peritoneal dissemination in the open surgery group appeared more frequent than that in the MIS group at pT4, although this result was not statistically significant (*p* = 0.251). However, other investigators have reported that the prognosis of pT4 disease in the laparoscopic surgery group tends to be worse than that in the open surgery group.^8^, ^10^ Great care should be taken to avoid contact with the tumor as far as possible during the surgery. In this study all MIS procedures were performed with maximum adherence to the no‐touch policy of our institution. In open surgery, the tumor is often directly touched by the surgeon's hands, in contrast with laparoscopic surgery, which may cause microscopic tumor spread through blood flow, lymphatic flow, and dissemination. In general, surgeons attempt not to grasp the tumor directly to prevent intraoperative micrometastasis. However, for larger tumors such attempts are more difficult. During open surgery for advanced tumors, surgeons need to handle the stomach and large tumors with their hands. During MIS of such tumors, the stomach is handled using laparoscopic forceps. Therefore, the possibility of micrometastasis during surgery is lower with MIS than with open surgery, which may explain the better prognosis in the MIS group in patients with a more advanced pT stage in this study.

This study had several limitations. First, this was a retrospective study conducted in a single institution, and we controlled for the differing clinical backgrounds via PSM analysis; however, potential biases could not be completely eliminated. Second, the sample in this study was relatively small. Third, the period for patient inclusion was long because open surgery accounted for the majority of surgeries before 2011, after which laparoscopic surgeries gradually increased. Fourth, the period for survival analysis was short, although the median follow‐up time for all patients was 52 mo. We believe that a larger sample would yield more reliable results.

In conclusion, in this study of older patients with gastric cancer the MIS group had fewer complications, less blood loss, and a shorter postoperative stay than the open surgery group. The long‐term outcomes did not significantly differ between the groups.

## AUTHOR CONTRIBUTIONS

Protocol/project development: Yamamoto, Omori. Data collection or management: Yamamoto, Omori, Masuike, Shinno, Hara, Sugase, Kanemura, Miyata. Data analysis: Yamamoto, Omori, Shinno, Hara. Article writing/editing: Yamamoto, Omori, Shinno, Hara, Takeno, Hirao, Miyata.

## FUNDING INFORMATION

None.

## CONFLICT OF INTEREST STATEMENT

The authors declare no conflicts of interest for this article.

## ETHICS STATEMENT

Approval of the research protocol: This study was approved by the Ethics Review Committee of Osaka International Cancer Institute (number #23151) and conforms to the provisions of the Declaration of Helsinki.

Informed Consent: N/A.

Registry and the Registration No. of the study/trial: N/A.

Animal Studies: N/A.

## Supporting information


Data S1.

## References

[1] Kenig J , Mastalerz K , Mitus J , Kapelanczyk A . The surgical Apgar score combined with comprehensive geriatric assessment improves short‐ but not long‐term outcome prediction in older patients undergoing abdominal cancer surgery. J Geriatr Oncol. 2018;9(6):642–648. 29859713 10.1016/j.jgo.2018.05.012

[2] Kim SW , Han HS , Jung HW , Kim KI , Hwang DW , Kang SB , et al. Multidimensional frailty score for the prediction of postoperative mortality risk. JAMA Surg. 2014;149(7):633–640. 24804971 10.1001/jamasurg.2014.241

[3] Sandini M , Pinotti E , Persico I , Picone D , Bellelli G , Gianotti L . Systematic review and meta‐analysis of frailty as a predictor of morbidity and mortality after major abdominal surgery. BJS Open. 2017;1(5):128–137. 29951615 10.1002/bjs5.22 PMC5989941

[4] Gupta PK , Gupta H , Sundaram A , Kaushik M , Fang X , Miller WJ , et al. Development and validation of a risk calculator for prediction of cardiac risk after surgery. Circulation. 2011;124(4):381–387. 21730309 10.1161/CIRCULATIONAHA.110.015701

[5] Kneuertz PJ , Pitt HA , Bilimoria KY , Smiley JP , Cohen ME , Ko CY , et al. Risk of morbidity and mortality following hepato‐pancreato‐biliary surgery. J Gastrointest Surg. 2012;16(9):1727–1735. 22760965 10.1007/s11605-012-1938-y

[6] Abete P , Cherubini A , Di Bari M , Vigorito C , Viviani G , Marchionni N , et al. Does comprehensive geriatric assessment improve the estimate of surgical risk in elderly patients? An Italian multicenter observational study. Am J Surg. 2016;211(1):76–83.e2. 26116322 10.1016/j.amjsurg.2015.04.016

[7] Stornes T , Wibe A , Endreseth BH . Complications and risk prediction in treatment of elderly patients with rectal cancer. Int J Colorectal Dis. 2016;31(1):87–93. 26298183 10.1007/s00384-015-2372-x

[8] Etoh T , Ohyama T , Sakuramoto S , Tsuji T , Lee SW , Yoshida K , et al. Five‐y survival outcomes of laparoscopy‐assisted vs open distal gastrectomy for advanced gastric cancer: the JLSSG0901 randomized clinical trial. JAMA Surg. 2023;158(5):445–454. 36920382 10.1001/jamasurg.2023.0096 PMC10018406

[9] Park YK , Yoon HM , Kim YW , Park JY , Ryu KW , Lee YJ , et al. Laparoscopy‐assisted versus open D2 distal gastrectomy for advanced gastric cancer: results from a randomized phase II multicenter clinical trial (COACT 1001). Ann Surg. 2018;267(4):638–645. 28187041 10.1097/SLA.0000000000002168

[10] Yu J , Huang C , Sun Y , Su X , Cao H , Hu J , et al. Effect of laparoscopic vs open distal gastrectomy on 3‐y disease‐free survival in patients with locally advanced gastric cancer: the CLASS‐01 randomized clinical trial. JAMA. 2019;321(20):1983–1992. 31135850 10.1001/jama.2019.5359 PMC6547120

[11] Tanaka R , Lee SW , Imai Y , Honda K , Matsuo K , Tashiro K , et al. Advantages of laparoscopic surgery for gastric cancer in elderly patients aged over 80 years: a propensity score matching analysis. World J Surg. 2021;45(9):2830–2839. 34019135 10.1007/s00268-021-06157-6

[12] Japanese Gastric Cancer Association . Japanese classification of gastric carcinoma: 3rd English Ed. Gastric Cancer. 2011;14:101–112. 21573743 10.1007/s10120-011-0041-5

[13] Brierley JD , Gospodarowicz MK , Wittekind C , editors. TNM classification of malignant tumours. 8th ed. Hoboken, NJ: Wiley Blackwell; 2017.

[14] Clavien PA , Barkun J , de Oliveira ML , Vauthey JN , Dindo D , Schulick RD , et al. The Clavien‐Dindo classification of surgical complications: five‐y experience. Ann Surg. 2009;250(2):187–196. 19638912 10.1097/SLA.0b013e3181b13ca2

[15] Charlson ME , Pompei P , Ales KL , MacKenzie CR . A new method of classifying prognostic comorbidity in longitudinal studies: development and validation. J Chronic Dis. 1987;40(5):373–383. 3558716 10.1016/0021-9681(87)90171-8

[16] Japanese Gastric Cancer Association . Japanese gastric cancer treatment guidelines 2018, 5th ed. Gastric Cancer. 2021;24:1–21. 32060757 10.1007/s10120-020-01042-y PMC7790804

[17] Yamamoto M , Omori T , Shinno N , Hara H , Fujii Y , Mukai Y , et al. Laparoscopic proximal gastrectomy with novel Valvuloplastic esophagogastrostomy vs. laparoscopic Total gastrectomy for stage I gastric cancer: a propensity score matching analysis. J Gastrointest Surg. 2022;26(10):2041–2049. 36038747 10.1007/s11605-022-05404-y

[18] Tanigawa N , Lee SW , Kimura T , Mori T , Uyama I , Nomura E , et al. The endoscopic surgical skill qualification system for gastric surgery in Japan. Asian J Endosc Surg. 2011;4(3):112–115. 22776273 10.1111/j.1758-5910.2011.00082.x

[19] Honda M , Kumamaru H , Etoh T , Miyata H , Yamashita Y , Yoshida K , et al. Surgical risk and benefits of laparoscopic surgery for elderly patients with gastric cancer: a multicenter prospective cohort study. Gastric Cancer. 2019;22(4):845–852. 30539321 10.1007/s10120-018-0898-7

[20] Kim KH , Kim MC , Jung GJ . Is the rate of postoperative complications following laparoscopy‐assisted gastrectomy higher in elderly patients than in younger patients? World J Surg Oncol. 2014;12:97. 24736010 10.1186/1477-7819-12-97 PMC3990240

[21] Yamamoto M , Shimokawa M , Kawano H , Ohta M , Yoshida D , Minami K , et al. Benefits of laparoscopic surgery compared to open standard surgery for gastric carcinoma in elderly patients: propensity score‐matching analysis. Surg Endosc. 2019;33(2):510–519. 30030615 10.1007/s00464-018-6325-7

[22] Shimada S , Sawada N , Oae S , Seki J , Takano Y , Ishiyama Y , et al. Safety and curability of laparoscopic gastrectomy in elderly patients with gastric cancer. Surg Endosc. 2018;32(10):4277–4283. 29602987 10.1007/s00464-018-6177-1

[23] Fujisaki M , Shinohara T , Hanyu N , Kawano S , Tanaka Y , Watanabe A , et al. Laparoscopic gastrectomy for gastric cancer in the elderly patients. Surg Endosc. 2016;30(4):1380–1387. 26123337 10.1007/s00464-015-4340-5

[24] Ueno D , Matsumoto H , Kubota H , Higashida M , Akiyama T , Shiotani A , et al. Prognostic factors for gastrectomy in elderly patients with gastric cancer. World J Surg Oncol. 2017;15(1):59. 28284210 10.1186/s12957-017-1131-6 PMC5346248

[25] Zurlo IV , Pozzo C , Strippoli A , Mignogna S , Basso M , Vivolo R , et al. Safety and efficacy of a first‐line chemotherapy tailored by G8 score in elderly metastatic or locally advanced gastric and gastro‐esophageal cancer patients: a real‐world analysis. Geriatrics (Basel). 2022;7(5):107. 36286210 10.3390/geriatrics7050107 PMC9601931

[26] Lu J , Huang CM , Zheng CH , Li P , Xie JW , Wang JB , et al. Short‐ and long‐term outcomes after laparoscopic versus open total gastrectomy for elderly gastric cancer patients: a propensity score‐matched analysis. J Gastrointest Surg. 2015;19(11):1949–1957. 26268957 10.1007/s11605-015-2912-2

[27] Zong L , Wu A , Wang W , Deng J , Aikou S , Yamashita H , et al. Feasibility of laparoscopic gastrectomy for elderly gastric cancer patients: meta‐analysis of non‐randomized controlled studies. Oncotarget. 2017;8(31):51878–51887. 28881697 10.18632/oncotarget.16691 PMC5584298

[28] Omori T , Fujiwara Y , Moon J , Sugimura K , Miyata H , Masuzawa T , et al. Comparison of single‐incision and conventional multi‐port laparoscopic distal gastrectomy with D2 lymph node dissection for gastric cancer: a propensity score‐matched analysis. Ann Surg Oncol. 2016;23(Suppl 5):817–824. 27510844 10.1245/s10434-016-5485-8

[29] Omori T , Fujiwara Y , Yamamoto K , Yanagimoto Y , Sugimura K , Masuzawa T , et al. The safety and feasibility of single‐port laparoscopic gastrectomy for advanced gastric cancer. J Gastrointest Surg. 2019;23(7):1329–1339. 30187335 10.1007/s11605-018-3937-0

[30] Omori T , Yamamoto K , Hara H , Shinno N , Yamamoto M , Fujita K , et al. Comparison of robotic gastrectomy and laparoscopic gastrectomy for gastric cancer: a propensity score‐matched analysis. Surg Endosc. 2022;36(8):6223–6234. 35229214 10.1007/s00464-022-09125-w

