# Supplementary material for: Minimally invasive surgery versus open gastrectomy for older patients with gastric cancer: A propensity score‐matching analysis
Source: Ann Gastroenterol Surg. 2024 Jul 15;9(1):69–78. doi: 10.1002/ags3.12842 (PMC11693573; doi:10.1002/ags3.12842)

**SUPPORTING INFORMATION**

**Supplementary Table 1 Recurrence pattern in pT4 before and after matching**

|  | Before matching |  |  |  | After matching |  |  |
| --- | --- | --- | --- | --- | --- | --- | --- |
|  | Open (n = 49) | MIS (n = 31) | *P* |  | Open (n = 26) | MIS (n = 29) | *P* |
| Recurrence | 28 (57.1%) | 8 (25.8%) | 0.006 |  | 16 (61.5%) | 8 (27.6%) | 0.011 |
| Peritoneal dissemination | 16 (32.7%) | 7 (22.6%) | 0.332 |  | 10 (38.5%) | 7 (24.1%) | 0.251 |
| Lymph nodes | 2 (4.1%) | 0 (0%) | 0.255 |  | 2 (7.7%) | 0 (0%) | 0.128 |
| Liver | 3 (6.1%) | 0 (0%) | 0.160 |  | 2 (7.7%) | 0 (0%) | 0.128 |
| Lungs | 1 (2.0%) | 0 (0%) | 0.424 |  | 0 (0%) | 0 (0%) | - |
| Local | 3 (6.1%) | 1 (3.2%) | 0.563 |  | 2 (7.7%) | 1 (3.4%) | 0.489 |
| Bone | 2 (4.1%) | 0 (0%) | 0.255 |  | 1 (3.8%) | 0 (0%) | 0.287 |
| Others | 3 (6.1%) | 1 (3.2%) | 0.563 |  | 1 (3.8%) | 1 (3.4%) | 0.937 |

*Open*, open surgery, *MIS*, minimally invasive surgery.

**Supplementary Figures**

Supplementary Figure 1. Comparison of survival curves between open surgery and MIS for disease-specific survival according to pT stage. *Open*, open surgery, *MIS*, minimally invasive surgery, *DSS*, disease-specific survival


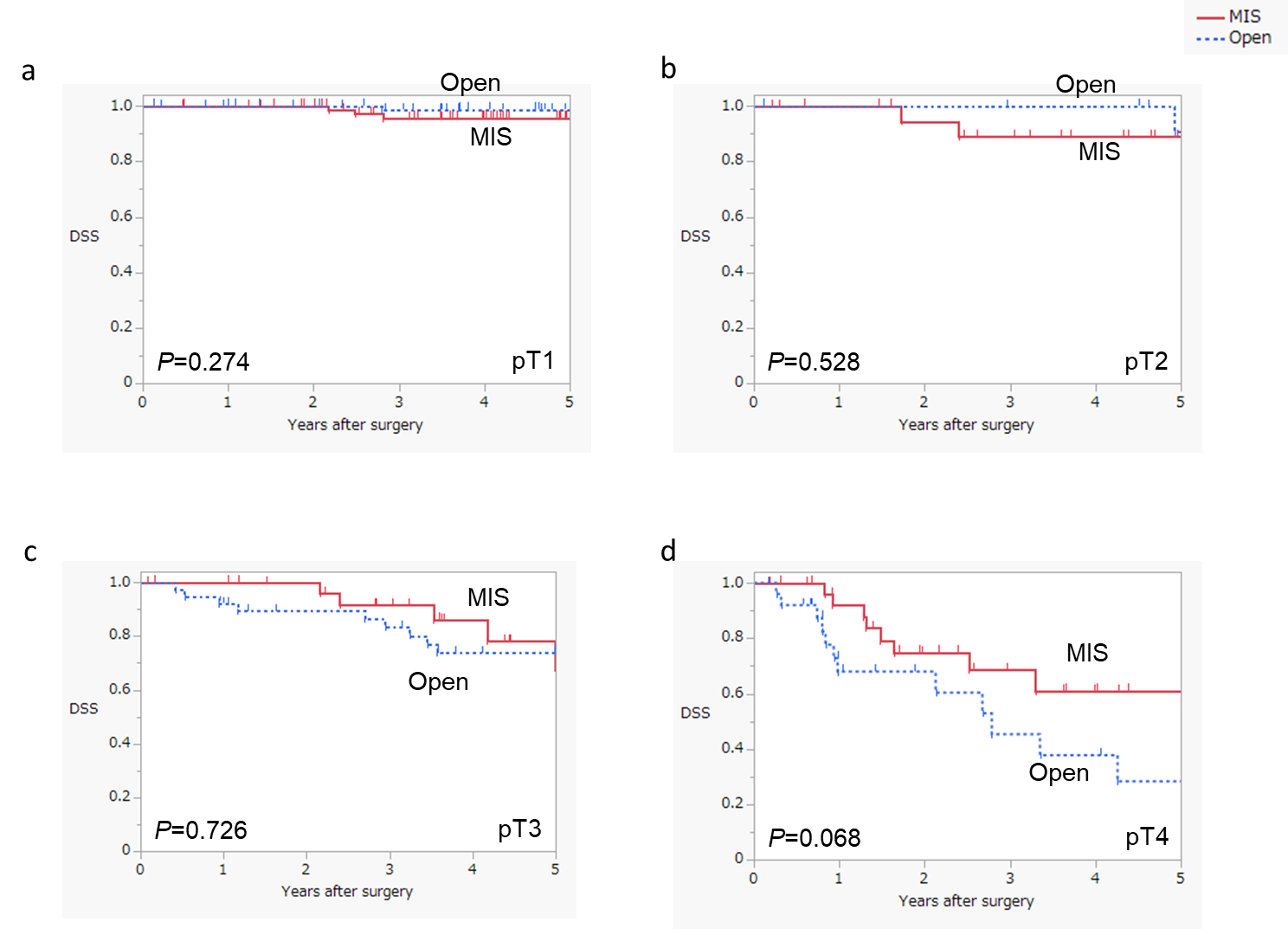


Supplementary Figure 2. Comparison of survival curves between open surgery and MIS for overall survival according to pN stage. *Open*, open surgery, *MIS*, minimally invasive surgery, *OS*, overall survival


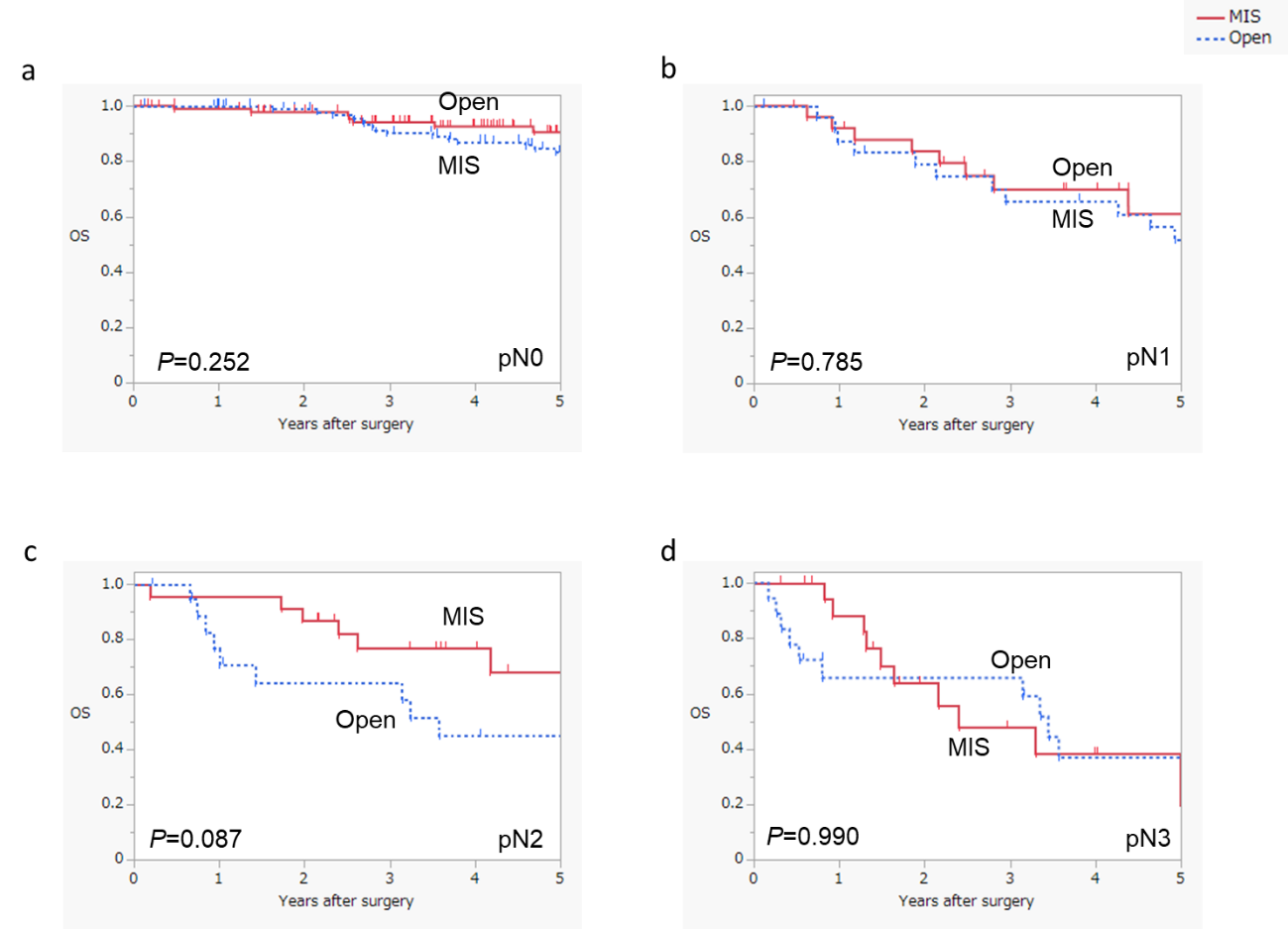


Supplementary Figure 3. Comparison of survival curves between open surgery and MIS for recurrence-free survival according to pN stage. *Open*, open surgery, *MIS*, minimally invasive surgery, *RFS*, recurrence-free survival


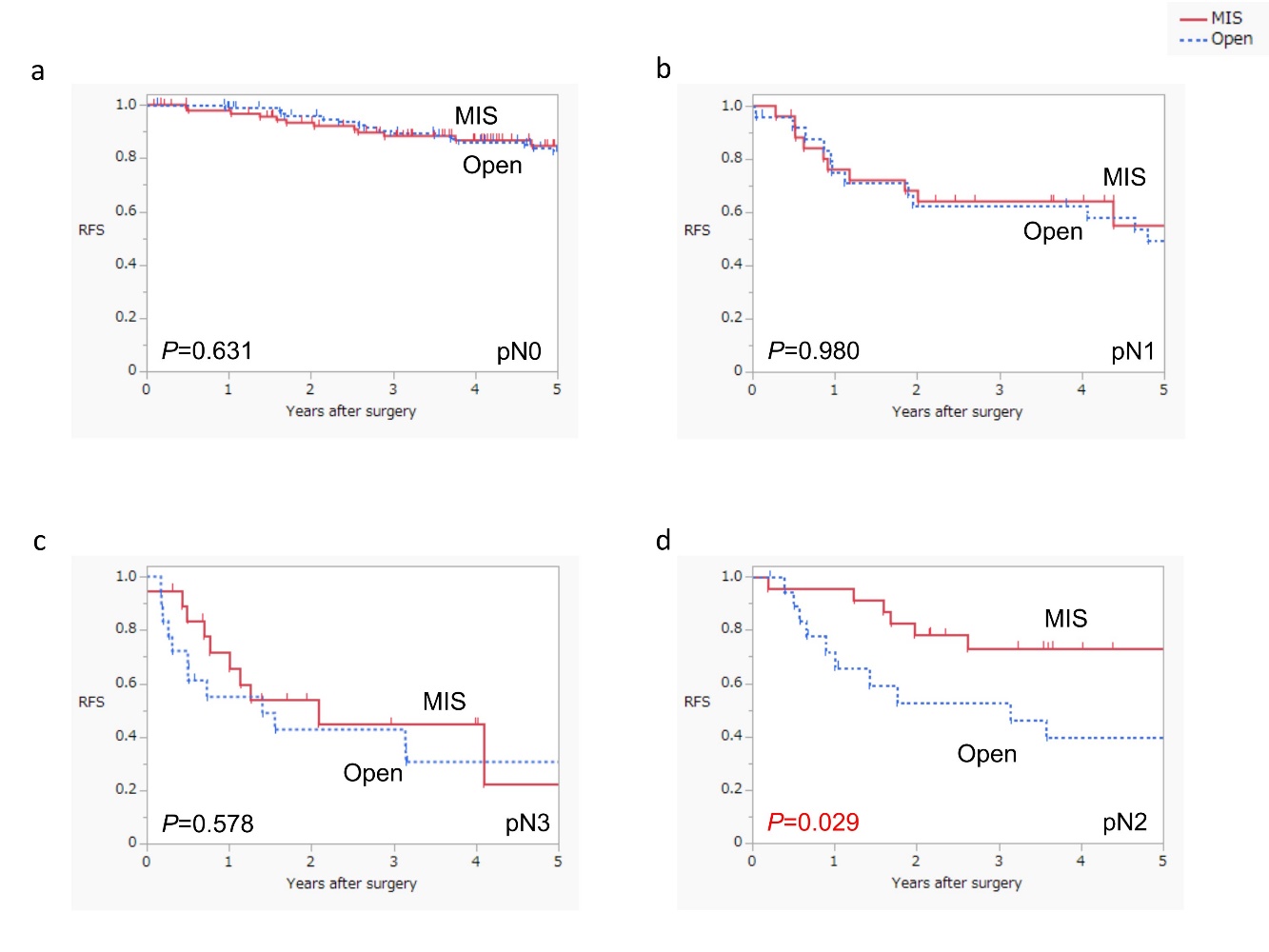


Supplementary Figure 4. Comparison of survival curves between open surgery and MIS for disease-specific survival. according to pN stage. *Open*, open surgery, *MIS*, minimally invasive surgery, *DSS*, disease-specific survival


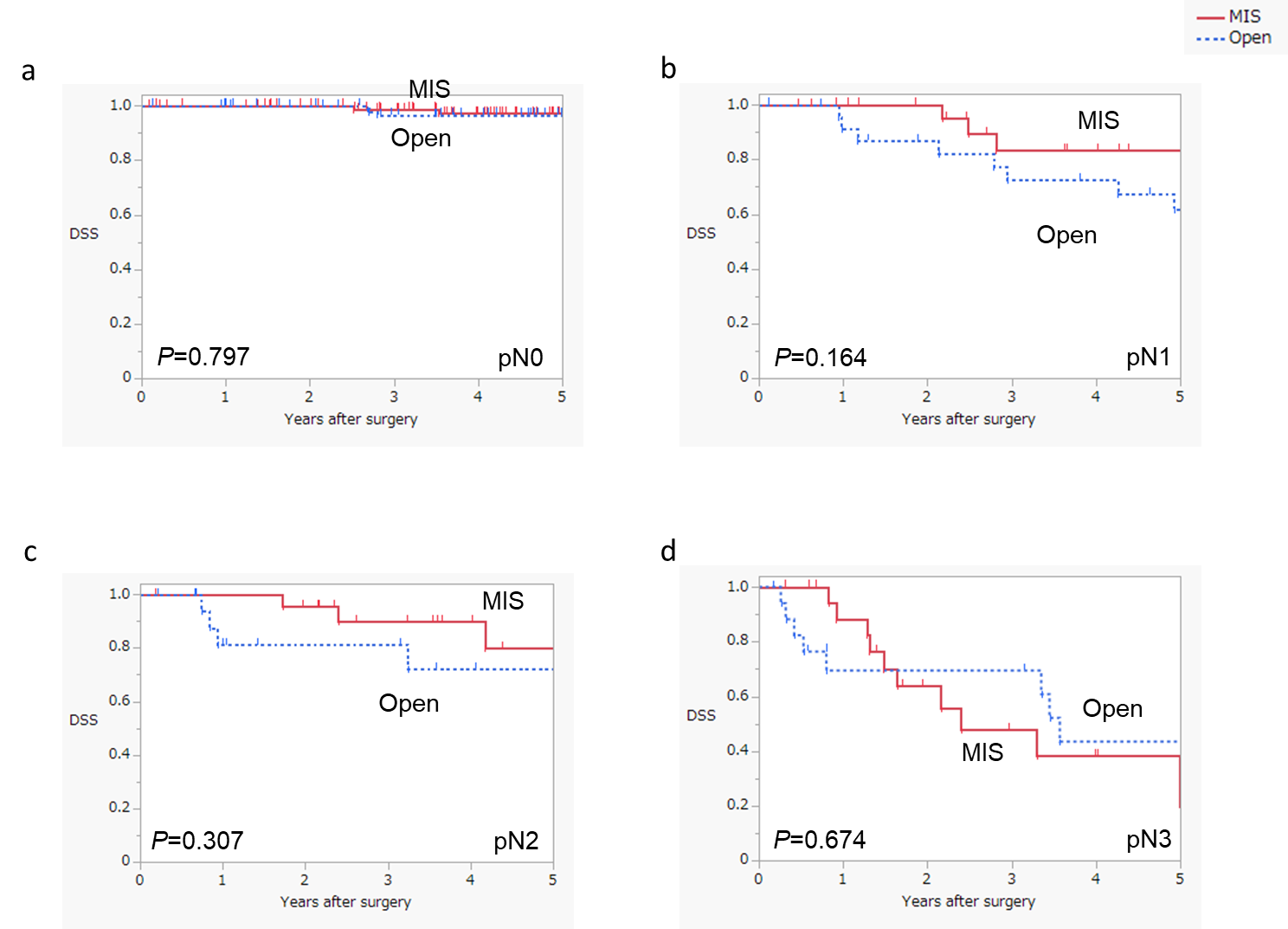


Supplementary Figure 5. Comparison of survival curves between open surgery and MIS for overall survival according to pathological stage. *Open*, open surgery, *MIS*, minimally invasive surgery, *OS*, overall survival


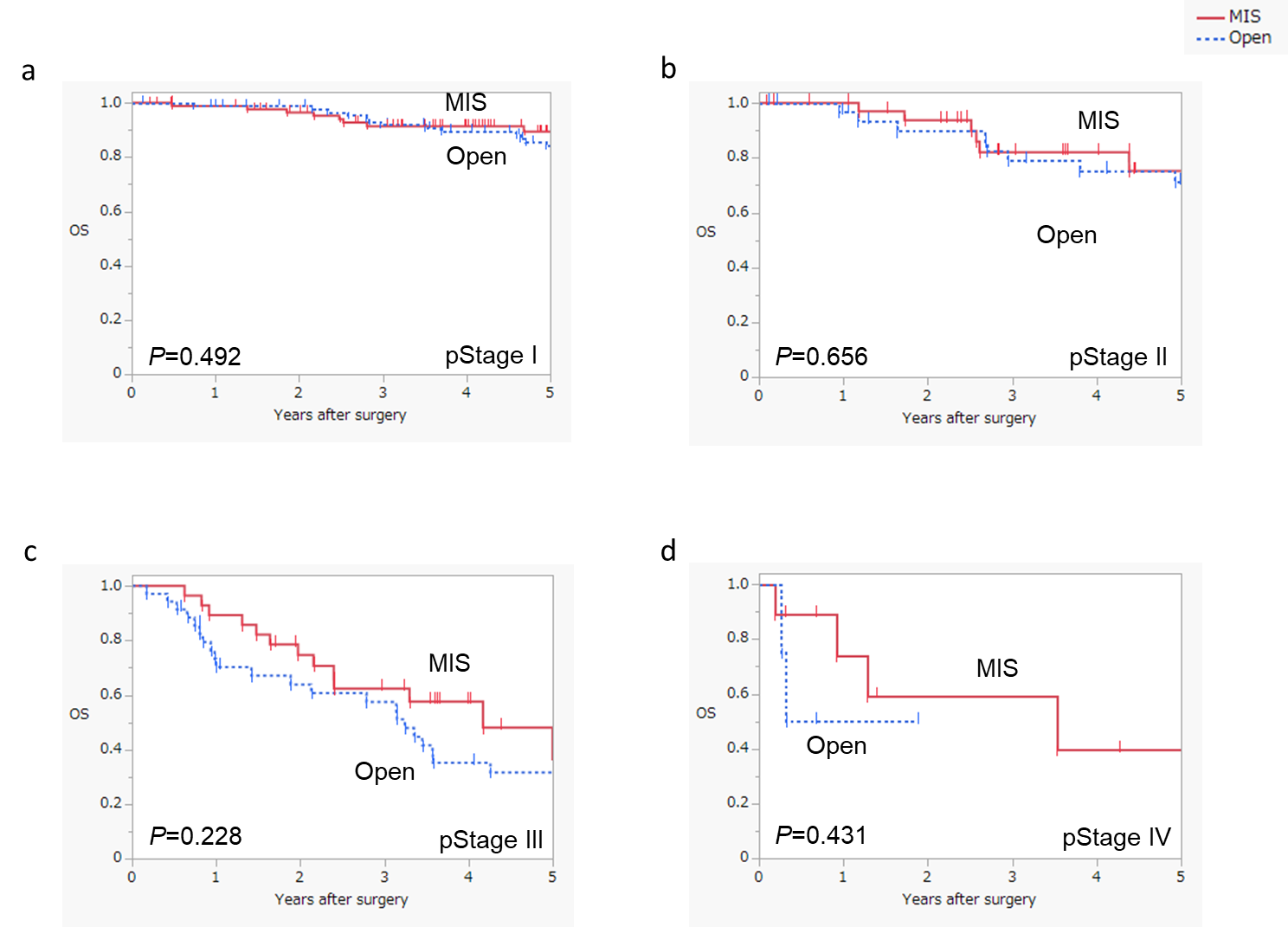


Supplementary Figure 6. Comparison of survival curves between open surgery and MIS for recurrence-free survival according to pathological stage. *Open*, open surgery, *MIS*, minimally invasive surgery, *RFS*, recurrence-free survival


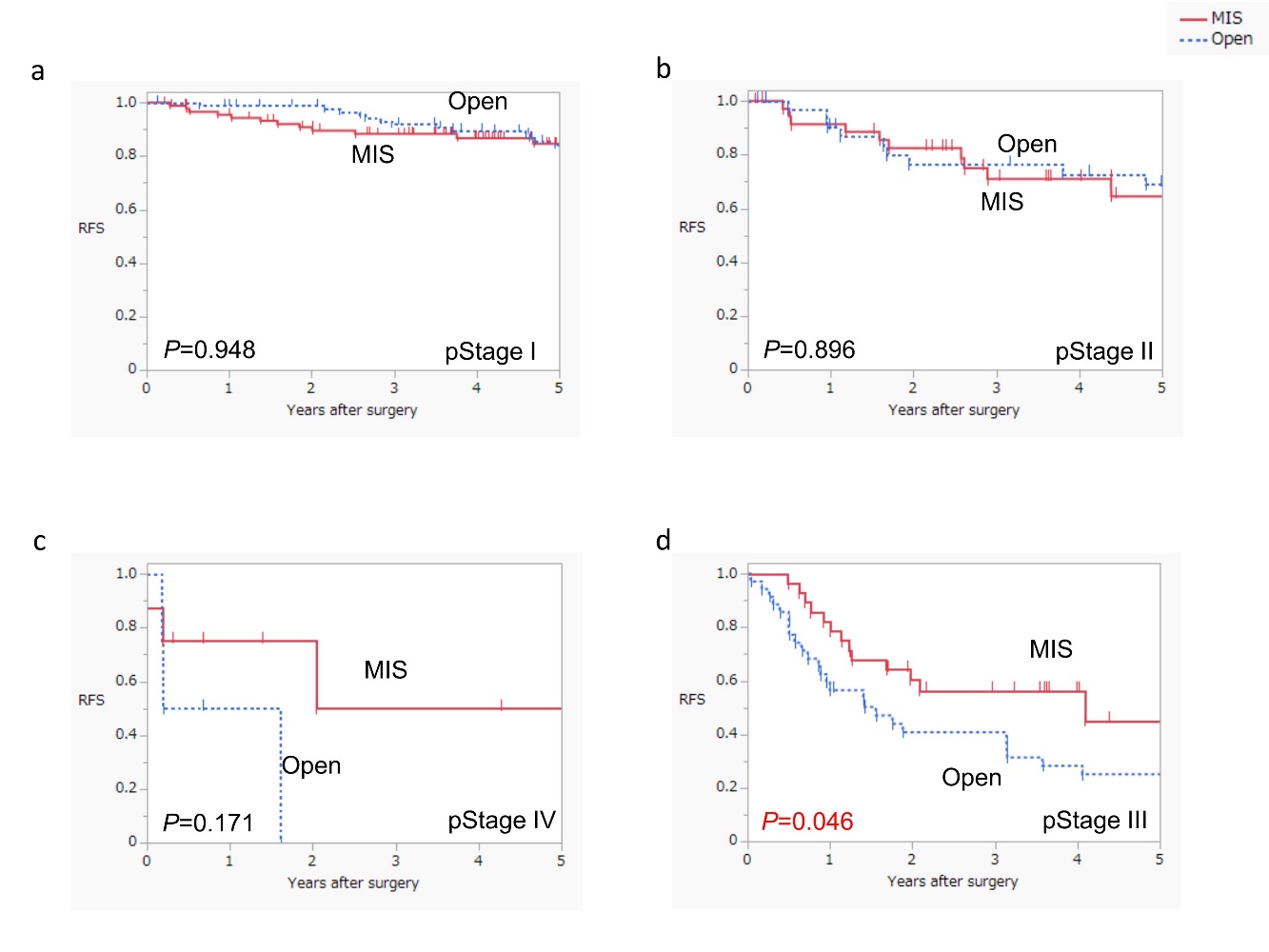


Supplementary Figure 7. Comparison of survival curves between open surgery and MIS for disease-specific survival according to pathological stage. *Open*, open surgery, *MIS*, minimally invasive surgery, *DSS*, disease-specific survival


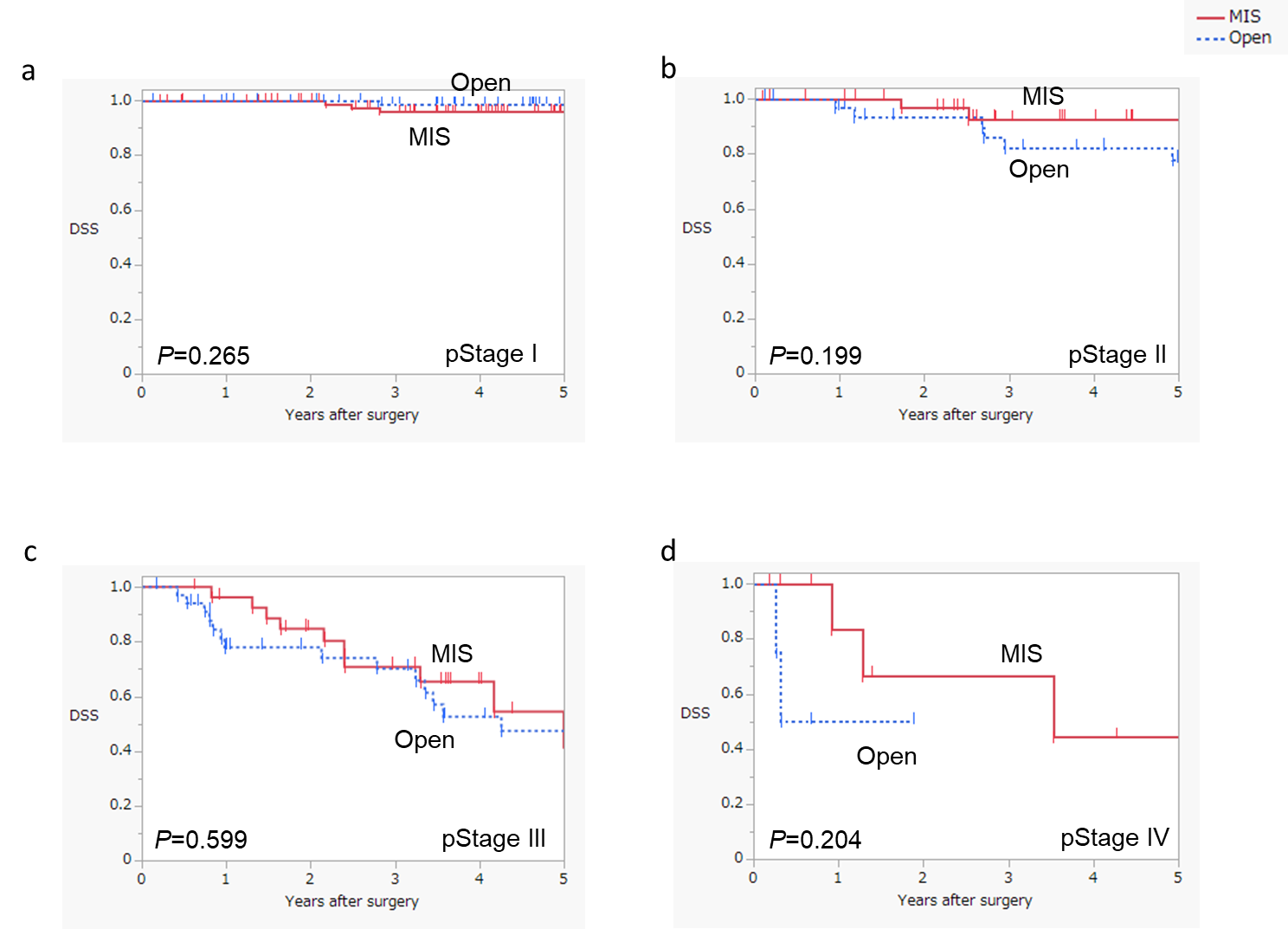

Supplement: Supplementary file 1 — Data S1. [file AGS3-9-69-s001.zip › ags3_12842_supp info.docx]
